# Evaluation of a ketogenic diet for improvement of neurological recovery in individuals with acute spinal cord injury: study protocol for a randomized controlled trial

**DOI:** 10.1186/s13063-020-04273-7

**Published:** 2020-05-04

**Authors:** Aynur Demirel, Jia Li, Casey Morrow, Stephen Barnes, Jan Jansen, Barbara Gower, Keneshia Kirksey, David Redden, Ceren Yarar-Fisher

**Affiliations:** 1 Faculty of Physical Therapy and Rehabilitation, Hacettepe University, Samanpazari, 06100 Ankara, Turkey; 2 Department of Physical Medicine and Rehabilitation, UAB School of Medicine, Spain Rehabilitation Center, 1717 6th Avenue South, Birmingham, AL 35233 USA; 3 Department of Cell, Developmental, and Integrative Biology, UAB School of Medicine, 1918 University Blvd, MCLM 680, Birmingham, AL 35233 USA; 4 Department of Pharmacology and Toxicology, The University of Alabama at Birmingham, Birmingham, AL 35294 USA; 5 Division of Acute Care Surgery, The University of Alabama at Birmingham, Birmingham, AL 35233 USA; 6 Department of Nutrition Sciences, UAB School of Health Professions, 1675 University Blvd., Webb 624C, Birmingham, AL 35294 USA; 7 Department of Biostatistics, The University of Alabama at Birmingham, 1720 2nd Avenue South, Birmingham, AL 35294 USA

**Keywords:** Spinal cord injury, Diet, Ketogenic, Proteomics, Nutrition therapy, High-protein diet, Low-carbohydrate diet

## Abstract

**Background:**

Therapies that significantly improve the neurological and functional recovery of individuals with spinal cord injury (SCI) are still urgently needed. The ketogenic diet (KD) has been shown to improve forelimb motor function in an SCI rat model, likely by reducing inflammation and cell death in the spinal cord. Furthermore, our recent pilot study in patients with SCI showed that, compared with a standard hospital diet (SD), 5 weeks of KD started during acute care improved upper extremity motor function and reduced serum levels of a neuroinflammatory blood protein. The primary goals of the current study are to: 1) show the safety and feasibility of administering a KD during acute care for SCI; 2) determine if consuming 5 weeks of a KD significantly improves motor and sensory functions, functional independence and glycemic control; and 3) quantify serum biomarkers that are linked to improvements in neurological recovery and functional independence via targeted proteomics.

**Methods/design:**

In a single-masked, longitudinal, randomized, parallel-controlled study, a total of 60 eligible, acutely traumatic spinal cord injured (cervical 5 to thoracic 12) participants ranging in age from 18 to 60 years with American Spinal Injury Association impairment scale (AIS) grades A–C (AIS-A, sensorimotor complete; AIS-B, sensory incomplete/motor complete; and AIS-C, nonfunctional motor incomplete) are being enrolled. Neurological and functional examinations, resting energy expenditure, blood, urine, and stool collections, and protein analyses related to neurological recovery will be performed within 72 h of injury (baseline measure) and repeated after 5 weeks of KD or SD (discharge measure). We anticipate a completion rate of 80% with a total of 48 participants.

**Discussion:**

Intervention with a more neuroprotective diet during acute care of SCI can be implemented anywhere in the world at low cost and without major regulatory hurdles. Better functional recovery will lead to a better quality of life and long-term health outcomes in individuals with SCI. While this study targets SCI, if successful it has the potential to improve neurological outcomes for individuals with various traumatic injuries.

**Trial registration:**

NCT03509571 Registered on April 28, 2018.

## Background

Despite advances in understanding the basic neurobiology of spinal cord injury (SCI), the development of therapeutic strategies for the acutely injured spinal cord has been agonizingly slow. Thus, SCI remains a significant cause of disability and mortality. SCI in humans not only leads to the well-recognized loss of motor and sensory function, but also the less-studied multiple secondary complications (i.e., bladder and bowel dysfunction, metabolic disorders and cardiovascular disease) caused by extreme physical inactivity due to paralysis [1–3]. These secondary complications of SCI contribute to the greatly reduced lifespan of these patients, which is rarely emphasized. While the field has made large strides in improving the survival of individuals with SCI in the acute 2-year phase after injury (40% reduction in mortality), there have been no gains in lifespan in the chronic situation over the last 20 years [4]. Furthermore, individuals with SCI who survive beyond the first 2 years after injury still die on average 13–25 years earlier than able-bodied people, depending on the level and severity of injury [4–6]. The current paradigm holds that better neurological recovery during the acute period will lead to a longer, more active and independent life for individuals with SCI. In addition, individuals with improved neurological recovery are expected to better manage problems such as spasticity, weight gain and chronic pain, and to be less likely to experience feelings of anxiety, loneliness and depression.

After the initial trauma of SCI, cell death and tissue loss continue for several weeks [7], a window in which one could effectively intervene with neuroprotective strategies. During this time, an escalating cycle of glutamate excitotoxicity, inflammation and neuronal apoptosis leads to progressive degeneration in the spinal cord [8]. Currently, the only approach taken to restrict secondary damage after SCI is surgical decompression and the administration of methylprednisolone. However, the recommendations to use methylprednisolone have fluctuated from “standard of care” to “optional” to “not recommended” and currently back to “optional” in the US and Canada since it has been shown to offer merely marginal benefit in select patient groups and increase the risk of major complications—in particular, gastrointestinal ulcers/bleeding during the acute stage of SCI [7]. In addition, several trials of neuroprotective treatments given in the acute phase after SCI have failed [9], while other approaches such as therapeutic hypothermia and administration of drugs targeting inflammation or glutamate excitotoxicity, for example, are still under investigation [8] and are not standard of care. This situation highlights the urgent need for new approaches for preventing secondary damage during the acute phase of SCI.

**Fig. 1:**
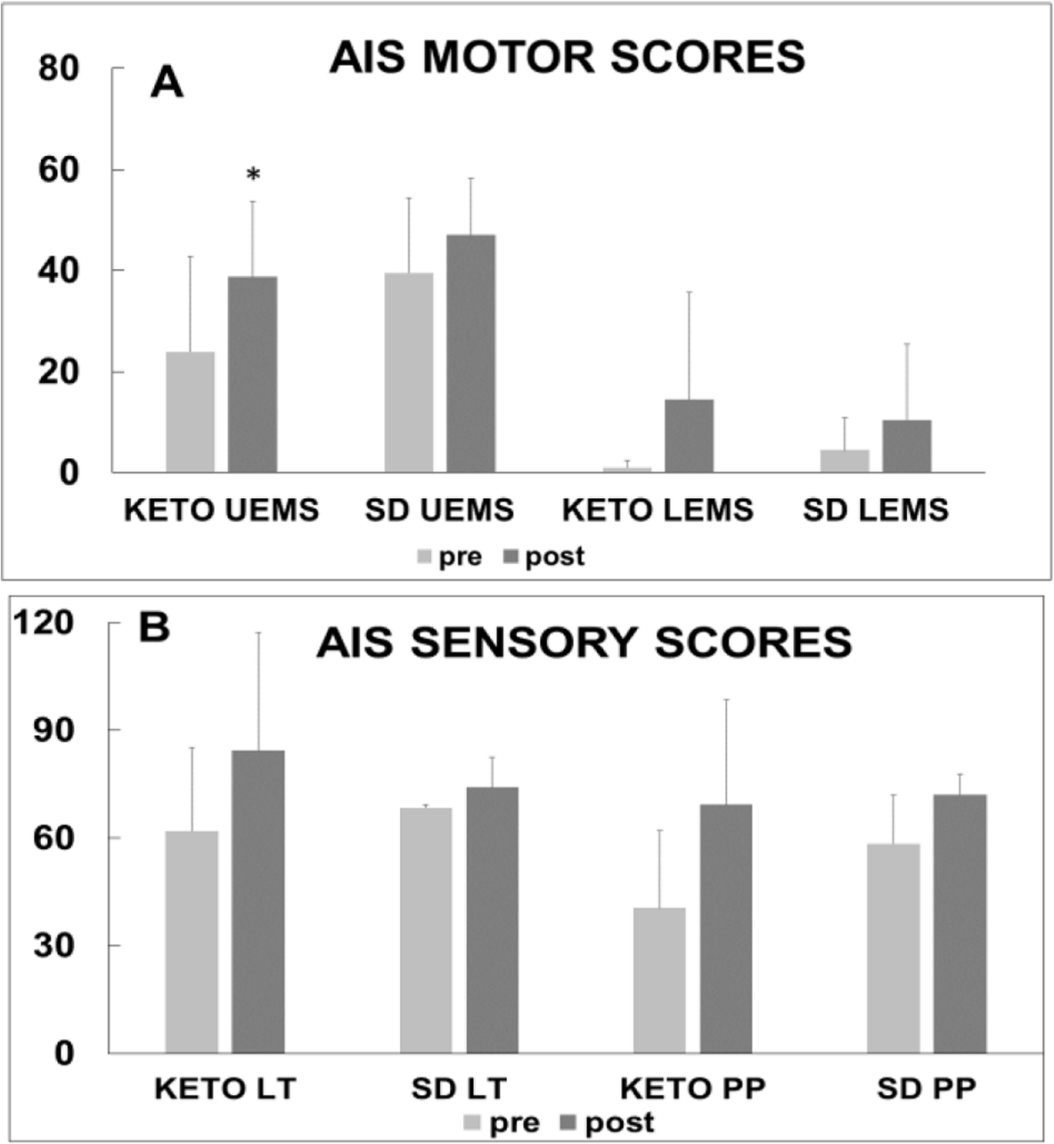
Effects of the ketogenic diet (KETO) versus standard diet (SD) on motor and sensory scores in patients with spinal cord injury. (**a**) American Spinal Injury Association Impairment Scale (AIS) upper extremity motor scores (UEMS) and lower extremity motor scores (LEMS). (**b**) AIS light touch (LT) and pin-prick (PP) sensory scores

Emerging evidence suggests that acute-phase hyperglycemia is a critical indicator of poor functional outcomes of SCI [10, 11]. The presence of hyperglycemia (≥126 mg/dl) on hospital admission (irrespective of past diabetes mellitus history) was found to be strongly associated with a lower probability of improvement in motor and sensory functions. Changes to diet might be the least invasive approach to recovery after neurotrauma. A wide variety of evidence suggests that the ketogenic diet (KD) could have beneficial disease-modifying effects in a broad range of neurological disorders [12–16]. The KD is composed of 80–90% fat, with carbohydrate and protein constituting the remainder of the intake. Energy is largely derived from the utilization of dietary fat. These fats are converted in the liver to ketone bodies β-hydroxybutyrate, acetoacetate and acetone, which provide the brain with an alternative energy source to glucose. The ability of a KD to ameliorate the diabetic state and help stabilize hyperglycemia has been repeatedly shown in human studies [17, 18]. In addition, a study by Streijger et al. showed the critical role of ketones in neuroprotection by starting a KD 4 h after cervical hemi-contusion in rats with SCI [19]. Consistent with the published effects of a KD in rat models of SCI and in humans with hyperglycemia, our randomized pilot feasibility trial [20] showed that, compared with a standard hospital diet (SD), 5 weeks of KD improved upper extremity motor scores (Fig. 1).

Although the exact mechanism by which the KD provides neuroprotection is not fully understood, three main effects on cellular energetics, mitochondria function and inflammation have been suggested to play a key role [21, 22]. Consequently, a KD may be optimal for maintaining a normal glycemic state, promoting inflammation regulation and rescuing mitochondrial function after SCI. Therefore, in this clinical trial, our purposes are to: *i*) show safety and feasibility to administer a KD in the acute stages of SCI; *ii*) determine if 5 weeks of KD versus SD significantly improves motor and sensory function, functional independence, glycemic control, and microbiome composition in patients with acute SCI; and *iii*) quantify serum biomarkers that are linked to improvements in neurological recovery and functional independence via targeted proteomics.

## Research methods/design

### Study design and participants

**Fig. 2:**
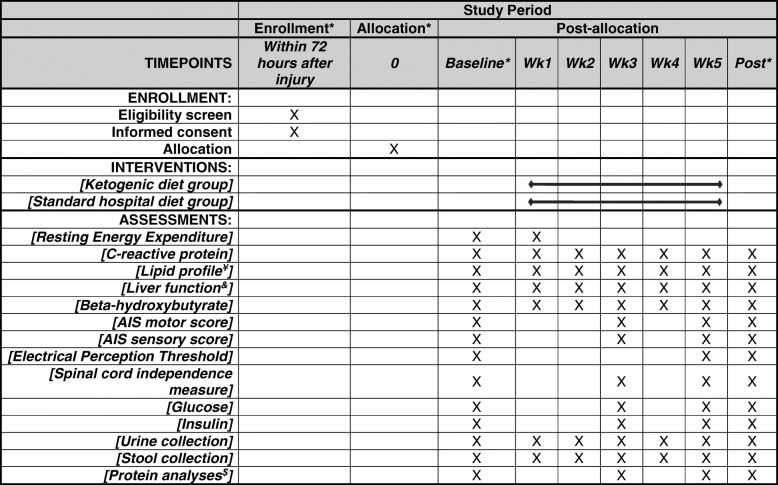
Standard Protocol Items: Recommendations for Interventional Trials (SPIRIT) schedule. *Allocation and baseline testing occur within 72 h of injury; postintervention assessments are performed prior to each patient’s discharge. ^¥^Including cholesterol, triglycerides, high-density lipoprotein cholesterol and calculated low-density lipoprotein cholesterol. ^&^As reflected by liver enzymes, alanine aminotransferase, aspartate transaminase, alkaline phosphatase, albumin, bilirubin and total protein. ^$^Serum fibrinogen, extracellular signal-regulated kinase 1/2, CD11b/CD18 integrin receptor, epidermal growth factor and receptor levels. AIS American Spinal Injury Association Impairment Scale, Wk week

**Fig. 3:**
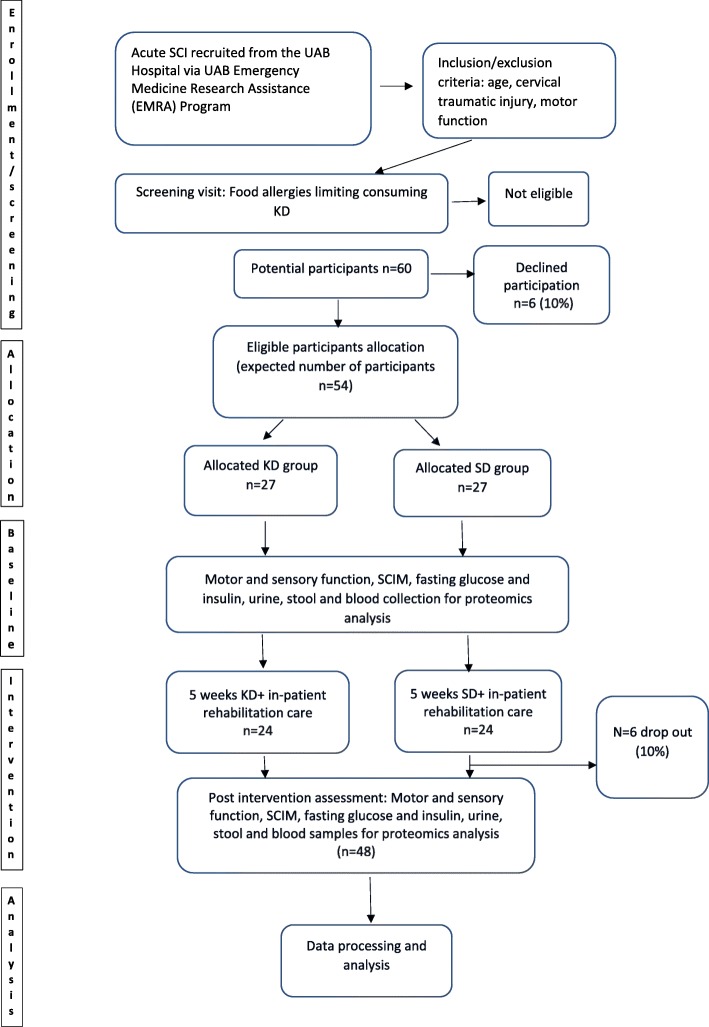
Flow diagram for the overall study design. KD ketogenic diet, SCI spinal cord injury, SCIM Spinal Cord Independence Measure, SD standard hospital diet, UAB University of Alabama at Birmingham

In a single-masked, longitudinal, randomized, parallel-controlled study design, eligible participants or legally authorized representative (if the participant is under mechanical ventilation) who provide informed consent to the principal investigator (PI) or assigned study coordinator are randomly assigned into a KD intervention group or an SD control group in a 1:1 ratio. Block randomization method with a block size of four is performed by the study statistician. A randomization list is generated, and the random assignments are placed into closed envelopes. All nurses performing blood draws, core facilities and research staff (technicians, students and trainees) analyzing the primary outcomes are blinded to group assignment. A Standard Protocol Items: Recommendations for Interventional Trials (SPIRIT) schedule is presented in Fig. 2 and the SPIRIT checklist is shown as Additional file 1. Outcome measures are performed at three time points: at baseline within 72 h of injury, before discharge from the acute care unit to the rehabilitation hospital, and at discharge from the rehabilitation hospital. Neurological examination via International Standards for Neurological Classification of SCI (ISNCSCI), commonly referred to as the American Spinal Injury Association Impairment Scale (AIS), electrical perception thresholds (EPTs) [23], functional examinations via the Spinal Cord Independence Measure (SCIM) [24, 25], resting energy expenditure (REE) and blood collection (for metabolic, targeted proteomics and safety outcomes) are performed within 72 h of injury, prior to starting enteral feeding (baseline measure). Following acute care, patients are transferred to the University of Alabama at Birmingham (UAB) Spain Rehabilitation Center (SRC) for inpatient rehabilitation care with an average length of stay of 20 days for patients with acute SCI. Accordingly, subsequent examinations are performed prior to transfer to the SRC (rehabilitation measure) and discharge from the SRC at week 5 (discharge measure). A summary of the data collection schedule for the outcomes is shown in Fig. 2, and the flow diagram for the overall study design is shown in Fig. 3.

The study is currently being conducted at the UAB Center for Clinical and Translational Science, UAB Diabetes Research Center Human Physiology Core, UAB Nutrition and Obesity Research Center Metabolism Core, UAB Microbiome Core and UAB Targeted Metabolomics and Proteomics Laboratory. Participants are considered eligible if they meet the following criteria: 1) are between the ages of 18 and 60 years; 2) have a diagnosis of traumatic SCI at the cervical or thoracic level (C5–T12); and 3) are classified as A (sensorimotor complete), B (sensory incomplete SCI; participants with sensory perception to S4 to S5, but no motor function preserved more than three segments below the cervical neurological level of injury), or C (nonfunctional motor incomplete; sensory and motor function are partially preserved below the neurological level of injury, but more than half the key muscles below the neurological level of injury have a muscle grade strength <3/5) on AIS. The exclusion criteria include: 1) patients with suspected traumatic central cord syndrome, because of the expected substantial spontaneous neurorecovery [26]; 2) pregnant women; 3) neurological (other than SCI), vascular, or cardiometabolic problems (e.g., hypertension or diabetes) that may limit function and interfere with testing procedures; 4) evidence of renal insufficiency or liver disease by history, physical examination and laboratory tests; 5) underlying pulmonary diseases; and 6) evidence of food allergies limiting consumption of a KD. Because of the differences in metabolic responses and nutritional needs among adults and children in the acute stages of traumatic injury, we only focus on adults with acute SCI to determine the effects of a KD on neurological recovery in this study; children (aged <18 years) are not eligible for this study.

The study population is expected to be representative of the demographics of the SCI patient population. Based on 2019 Spinal Cord Injury Facts and Figures, 78% of SCIs reported to the national database have occurred among males [27]. Therefore, we anticipate that, among those who participate in this study, 78% are male and 22% are female. SCI is more common in non-Hispanic white individuals (60.6%), followed by 21.9% in non-Hispanic black individuals, 12.8% with Hispanic origin, 0.7% Native Americans, 2.7% Asian, and 1.3% other; therefore, we expect that more non-Hispanic white individuals compared with other racial or ethnic groups will participate in this study.

### Recruitment

Participants are recruited from the UAB Hospital Trauma, Burns, and Surgical Critical Care (Acute Care) Unit. The PI is responsible for participant recruitment and trial coordination. Screening for the target participant pool is conducted by the UAB Emergency Medicine Research Assistance program. Participants are compensated US$100 for their time.

### Adequacy of the potential participant pool

The primary duty of the UAB Emergency Medicine Research Assistance program is to maintain a presence in the university hospital emergency department on a 24/7 basis. This program provides the research infrastructure necessary to carry out screening and enrollment in numerous clinical trials in the emergency department, which treats 100,000 people per year. The research assistants assist the main research study team in determining patient eligibility by confirming with the existing clinical staff the enrollment inclusion and exclusion criteria. They also notify key research personnel of study enrollment and provide information to the physicians regarding study status, eligibility requirements and general protocol information.

### Interventions/groups

#### The ketogenic diet group

This diet is a high-fat, low-carbohydrate diet (3:1 ratio of fat to carbohydrate and protein) that includes ~75% total energy as fat, ~20% as protein and ~5% as carbohydrate and fiber. Dietary fat sources focus on animal and vegetable (for vegetarians) fats. Dietary fat resources for vegan participants include nuts, seeds, avocados and coconut oil. Dietary protein sources include animal, plant and nut and seed proteins, and dietary carbohydrate resources include vegetarian and vegan sources. KetoCal®, a nutritionally complete, ready-to-feed ketogenic formula in a 3:1 ratio (fat to carbohydrate and protein), is provided by Nutricia for the patients who receive enteral nutrition. Nutricia is a global health company that has been the main provider of KDs for clinical trials, hospitals, health care professionals and communities around the world. Patients who are able to swallow receive liquid or solid high-fat meals that are prepared by the UAB research kitchen, with the overall energy amount determined according to REE assessed via indirect calorimetry and multiplied by an activity factor. REE is measured after a 12-h fast.

#### The standard diet group

Standard enteral tube feeding includes ~45–50% total energy as carbohydrate and fiber, ~30% total energy as fat and ~20% total energy as protein. When patients are able to receive liquid or solid feeding, they are expected to consume ~30% total energy as fat, ~25% as protein and ~45% as carbohydrate based on our preliminary results. Food is provided by UAB main hospital kitchen, and patients are allowed to choose options from the standard hospital menu, with the overall energy amount determined according to REE assessed via indirect calorimetry and multiplied by an activity factor.

REE measurements are performed in the patient’s hospital room. Participants lie supine on a comfortable bed with the head enclosed in a plexiglass canopy. After resting for 15 min, REE is measured for 30 min with a portable computerized, open-circuit, indirect calorimetry system with a ventilated canopy (Vmax ENCORE 29 N Systems; SensorMedics Corporation, Yorba Linda, CA, USA). Oxygen uptake and carbon dioxide production are measured continuously and values are averaged at 1-min intervals, and the REE is calculated from these data. The last 20 min of measurement is used for analysis.

### Inpatient rehabilitation care

Patients in both groups undergo an intensive rehabilitation program for about 5 weeks. Therapy is offered 5 days (a total of 15 h) per week. The standard of care for both groups includes: respiration therapy, bed mobility, transfers, wheelchair mobility skills, bowel and bladder management, tone and spasticity management, and skills for performing other activities of daily living. Rehabilitation protocols and treatment hours are recorded for each patient. The average duration of stay in the SRC is 30 days for individuals with tetraplegia (injury level C4–C7) and 21 days for individuals with paraplegia (injury level T1–S5).

## Outcome measures and analysis

The primary outcomes are as follows: 1) safety via biomarkers of systemic inflammation (C-reactive protein), lipids (total cholesterol, high-density lipoprotein cholesterol, low-density lipoprotein cholesterol and triglycerides), sodium, liver enzymes (alanine aminotransferase, aspartate transaminase, alkaline phosphatase, albumin, bilirubin, total protein, nutritional ketosis (β-hydroxybutyrate) and glycemic status (fasting blood glucose and insulin)); 2) neurological recovery (AIS) motor and sensory scores and EPTs; 3) functional independence (SCIM); 4) microbiome composition; and 5) serum biomarkers (targeted proteomics).

In compliance with goals of the National Institute of Neurological Disorders and Stroke common data elements initiative, we are utilizing portions of the SCI common data elements that are expected to increase the efficiency and effectiveness of our clinical research study and diet intervention, increase data quality, and facilitate data sharing and comparisons across studies. For data analyses and results reporting, we adhere to the guidelines for reporting results using the international spinal cord core dataset [28].

### Minimally acceptable intervention adherence rates

Adherence to diet will be closely monitored by the study team by measuring serum β-hydroxybutyrate levels once a week. A blood concentration of 0.5–3 mM (nutritional ketosis) will be considered safe and acceptable. Adherence will also be assessed using food records. The food record will be a simple checklist to indicate that supplied foods were consumed, with space to add the quantity of consumption and any deviations from the protocol. In addition, the study dietician will communicate directly with each participant at least weekly to improve adherence.

### Clinical procedures to measure safety

Fasting blood samples are collected to determine the levels of biomarkers of systemic inflammation, lipids, sodium, liver enzymes and glycemic status. In addition, blood samples are collected for measurement of blood ketone (β-hydroxybutyrate) levels three times a week, 2 h after the first meal of the day, to confirm nutritionally induced ketosis (blood concentration 0.5–3.0 mM) in the KD group. All serum samples will be obtained in the UAB Hospital Acute Care Unit and then stored in freezers within the PI’s (CY-F) research laboratory at −80°C until analyzed by core personnel. Levels of sodium, β-hydroxybutyrate, lipids, C-reactive protein, glucose and insulin are assessed via enzymatic assays in the Diabetes Research Center Human Physiology Core. The concentration of low-density lipoprotein cholesterol is calculated using the Friedewald formula. In addition, levels of liver enzymes are measured via enzymatic assays at the UAB Hospital Outreach Laboratory.

### Procedures to measure neurological recovery

The motor and sensory examinations are performed according to the ISNCSCI standards [29–31] and described by AIS. The scales used for the neurological tests for this proposal were selected because they are standard, widely accepted tools and can be readily administered in the acute SCI setting. The AIS Motor Index Score is used for measuring motor function. This index uses standard manual muscle testing on a six-point scale (graded 0–5). The maximum motor score for upper and lower extremity is 50. For the sensory examination, each dermatome is tested for both sharp (pin-prick) and light touch sensation and sensory status is graded on a three-point scale. Numerically, the sensory scores total 116 points.

EPTs are recorded according to established standards [23]. In brief, electrical stimuli are delivered at 3 Hz at key points within cervical dermatomes (C4 to C8). Stimulation commences at 0 mA and progressively increase at 0.1-mA intervals until the participant perceives a stimulus. The stimulus is then turned down until perception is lost. This process is repeated three times to generate an average EPT for each dermatome.

### Procedures to measure functional independence

The SCIM is used for measuring functional independence [24, 25] and is, at present, the only comprehensive rating scale that measures the ability of patients with SCI to perform everyday tasks according to their value for the patient. It requires no manual testing and the range of the total score is 0–100. Although one American Spinal Injury Association In-Step certified clinician performs all examinations, inter-rater reliability between the primary examiner and back-up examiners are assessed periodically as a quality assurance measure for consistency and a precautionary measure in the event that a back-up examiner might be required. Examiners are blinded to the diet assignment. Neurological examinations are routine both in the acute and inpatient care (in the SRC) facilities under the supervision of physiatrist.

### Procedures to determine gut microbiome composition

Microbiome collection and analysis are performed using established protocols [18]. Briefly, stool samples are collected in ParaPak vials (Meridian Biosciences, Inc., Cincinnati, OH, USA) and consequently diluted to 0.1 mg/ml in Cary-Blair medium for a total volume of 20 ml with 10% glycerol (by volume). Aliquots of 5 ml are stored at −80°C in cryovial tubes until DNA extraction. Stool bacterial DNA is extracted using a Zymo Research Fecal DNA isolation kit (Zymo Research, Irvine, CA, USA) as per the manufacturer’s instructions. Polymerase chain reaction is then used to amplify the V4 region of the 16S rRNA gene. The polymerase chain reaction products are then retrieved after separation on an agarose gel using electrophoresis, excised from the gel, and subsequently purified with a QIAquick Gel Extraction Kit (Qiagen, Germantown, MD, USA). The Illumina MiSeq DNA sequencing platform is used to sequence 250 base paired-end kits.

Microbiome analyses will be performed using the Quantitative Insights into Microbial Ecology bioinformatics software and QWRAP program as previously described [18]. Briefly, quality assessment and filtering of low-quality data will be performed using the FASTQC and FASTX toolsets, respectively. A combination of tools within the Quantitative Insights into Microbial Ecology software suite will be utilized for clustering reads into operational taxonomy units (uclust), taxa assignment (RDP classifier using the Greengenes 16S rDNA database) and, as necessary, alignment and phylogenetic inference (using PyNAST and FastTree). These procedures will allow us to quantitatively assess the microbiome population down to the genus, if not species, level. For comparative analyses, indices of alpha (Chao1, Observed species, Phylogenetic distance, Simpson’s index and Shannon’s index) and beta diversity (Bray–Curtis dissimilarity, unweighted UniFrac and weighted UniFrac) will be computed [18].

### Procedures to quantify serum biomarkers that may be linked to improvements in neurological recovery and functional independence

#### Sample processing

Serum samples are processed using disposable affinity-depletion cartridges (NorGen Biotek Corp.) that remove the major serum proteins albumin, α-antitrypsin, transferrin and haptoglobin [32]. Protein samples are evaporated and resuspended in 6 M urea, 100 mM Tris buffer at 10 mg/ml. Aliquots of the affinity-depleted serum are reduced using dithiothreitol (25 mM) at 50°C for 30 min followed by alkylation of free thiol groups with iodoacetamide (55 mM) for 30 min in the dark at room temperature. Solutions are then diluted 1/10 in double-distilled water (ddH_2_O) and overnight digestion is carried out using mass spectrometry (MS)-grade trypsin (0.2 μg/μl). The digests are evaporated to dryness in a Speedvac and then resuspended in 50 μl ddH_2_O with 0.1% formic acid.

#### Targeted protein analysis

Peptides representing fibrinogen, extracellular signal-regulated kinase 1/2, CD11b/CD18 integrin receptor and epidermal growth factor are selected from either preliminary data (fibrinogen) or using Skyline, a targeted proteomics software tool [33]. Predicted product ions from each peptide are used to set up a multiple reaction monitoring liquid chromatography tandem MS (LC-MS/MS) assay carried out on a SCIEX 6500 Qtrap using microflow LC. This instrument is very sensitive and is capable of detecting peptides in the amol range. The tryptic peptides are resolved on a 5–50% linear gradient of acetonitrile in 0.1% formic acid mobile phase before entering the electrospray ionization (ESI) interface of the mass spectrometer. Synthetic peptides will be used to obtain quantitative data. To obtain absolute quantitative data on the selected peptide(s), ^13^C/^15^N-isotopically labeled versions of the peptide(s) will be prepared [34].

#### Protein library generation

An aliquot (5 μl) of each digest is loaded onto a Nano cHiPLC 200 μm × 0.5 mm ChromXP C18-CL 3-μm 120-Å reverse-phase trap cartridge (Eksigent, Dublin, CA, USA) at 2 μl/min coupled to an Eksigent 415 LC system autosampler. After washing the cartridge for 10 min with 0.1% formic acid in ddH_2_O, the bound peptides are flushed onto a Nano cHiPLC column (200 μm ID× 15 cm ChromXP C18-CL 3 μm 120 Å; Eksigent) with a 100-min linear (5–50%) acetonitrile gradient in 0.1% formic acid at 1000 nl/min using an Eksigent Nano1D + LC. The column then is washed with 90% acetonitrile 0.1% formic acid for 5 min and re-equilibrated with 5% acetonitrile 0.1% formic acid for 15 min. A SCIEX 5600 TripleToF mass spectrometer (SCIEX, Toronto, Canada) is used to analyze the protein digest. The ionization spray voltage is set at 2300 V and the declustering potential set at 80 V. The ionization spray and curtain gases are set at 10 psi and 25 psi, respectively. The interface heater temperature is 120°C. In each 1.25-s duty cycle, eluted peptides are subjected to a 250-ms time-of-flight survey scan covering a range of 400–1250 m/z to determine the top 20 most intense ions for MS/MS analysis. Product ion time-of-flight scans at 50 ms are carried out to obtain the tandem mass spectra of the selected parent ions over a m/z range of 400–1000. Spectra are centroided and de-isotoped by Analyst software, version 1.7 TF (SCIEX). A β-galactosidase trypsin digest is used to establish and confirm the mass accuracy of the mass spectrometer. The MS/MS data are processed to provide protein identifications using an in-house Protein Pilot 4.5 search engine (SCIEX) using the UniProt *Homo sapiens* protein database and a trypsin digestion parameter. Proteins are included in the SWATH (sequential window acquisition of all theoretical mass spectra) library on the criteria of having at least two peptides detected with a confidence score of 95% or greater using the paradigm method imbedded in the Protein Pilot software.

#### SWATH-MS analysis

Individual samples are analyzed by the same nanoLC-ESI-MS/MS method described above. SWATH data [35] are collected in 1.8-s duty cycles—a 200-ms time-of-flight survey scan from m/z 400–1250 followed by 32, 50-ms successive tandem mass spectra using 25 m/z mass windows. The MS/MS data are deconvoluted and processed using PeakView™ 1.2 with the SWATH application (SCIEX). This software uses a pregenerated protein identification library to construct individual peptide signatures with fragmentation patterns after analyses for protein identifications. In addition, the areas of the peptide peaks in the MS/MS spectra are used for protein quantification. Identification of proteins associated with the phenotypes being studied will be carried out by univariate (volcano plots) and multivariate (principal components and partial least squares-discriminant analysis) analyses and associated heat-maps.

### Power calculations

Power calculations were performed using nQuery Advisor + nTerim 3.0, and assume a two-sided statistical test and a significance level of 5%. We obtained estimates of the standard deviation for the motor score of 23.5 and for the light touch score of 33 (both based on our preliminary data). With a final sample size of 24 participants per group, and also assuming a two-group *t* test and the prior assumptions, we have 80% power to detect between-group differences of 19.5 in the motor score and 27.3 in the light touch score as being statistically significant. With a final sample size of 24 participants per group, and assuming a paired *t* test and the prior assumptions, we have 80% power to detect within-group changes of 14.1 in the motor score and 19.8 in the light touch score as being statistically significant. All of these differences are approximately at the same levels as those that Kramer et al. detected as being statistically significant and clinically meaningful [36]. We believe that these estimates are conservative since we are performing our primary statistical analyses for between-group and within-group comparisons simultaneously using statistical methods that are more sophisticated than those that are assumed here. Given our history in SCI patient recruitment and the large SCI patient base at the UAB SRC, we fully expect to meet our recruitment goal.

### Randomization and blinding

Randomization is performed using the block randomization method, with a block size of four (study statistician). A randomization list is generated, and the random assignments placed into closed envelopes. Each study participant opens one of these envelopes to learn of their group assignment. Study patients will inevitably know their group assignment as we are limited by the difficulties of inadequate masking of the ketogenic diet, which is very restrictive and requires avoidance of usual foods like bread, pasta, rice, potatoes and a wide variety of fruits. In order to avoid a potential placebo effect or a participant biasing results due to knowledge of their diet, during the consenting process we simply explain that the study is designed to examine the impact of KD and SD on outcomes. In addition, individuals performing clinical and laboratory tests and the study statistician were blinded to the study interventions.

### Data management

To protect privacy, each study participant is assigned a unique four-digit identification number that cannot be traced to any protected health information (PHI). All data forms, participant information, and biological specimens are coded using these identification numbers. No personally identifiable information (PHI) appears on these materials; instead, the keys linking participants’ identities to their unique identification numbers are stored separately in a secure software system designed for clinical trials which meets both HIPAA and 21 CFR Part 11 requirements.

All study data are managed in a central database using REDCap, a secure web-based software system designed for clinical trials which meets both HIPAA and 21 CFR Part 11 requirements. All questionnaires and daily surveys are coded into REDCap and administered electronically. Data from serum assays, REE measurements, and sensory measures via dermatomal somatosensory evoked potentials and EPTs are output from the respective technical equipment in electronic form and then uploaded directly into the REDCap database. However, data on adverse events that are collected by the medical team are recorded on paper forms, stored in the clinical research unit, and then manually entered into the REDCap database. Neurological data (assessed by ISNCSCI standards) and food intake data are captured on paper forms and double entered into the database.

Paper documents are scanned and saved on UAB’s secure network and/or stored in locked cabinets in the research coordinator’s office. The PI and postdoctoral trainee periodically check the database for missing data and document all such data and the reasons for absence. The study statistician also periodically performs a quality check of the database, which includes error checks using expected ranges for data values. After the study has completed, all data quality will be checked by the statistician, PI, and the postdoctoral trainee in year 3; tasks are divided among the three personnel according to their respective responsibilities. In compliance with UAB Institutional Review Board (IRB) policies, all data and records will be kept for at least 3 years after the study is completed; all PHI for participants will be deleted 3 years after the trial is completed, while the deidentified final dataset will be retained indefinitely and published online for other scientists to benefit from.

### Statistical analysis

Statistical analyses for neurological recovery will include comparisons of means of motor and sensory function, functional independence and serum measurements (e.g., glucose, insulin, alanine aminotransferase and aspartate transaminase) between the two groups, as well as comparisons within each group of the changes from baseline to rehabilitation to discharge (which should take approximately 5 weeks). Descriptive statistics, including measures of central tendency (sample mean, sample median) and dispersion (variance) will be calculated for each group. For safety outcomes, instead of comparing the two groups to the established standard safety values, we will compare circulating levels of proposed outcomes at the various time points among the two groups. If there are no significant group differences in these outcomes, we will conclude that the KD intervention is safe. We will also calculate 95% confidence intervals for the mean group differences for these outcomes as an additional check on safety and feasibility. The primary method of analysis will be analysis of covariance (ANCOVA), regressing the 5-week outcomes (safety, motor and sensory recovery, functional independence, and glycemic state) on the randomly assigned diet, baseline values of the measures, and other covariates such as age and sex. Repeated measures ANCOVA will also be used. Statistical assumptions will be assessed using box plots, residual plots and normal probability plots. Secondary analyses, accounting for all time points, will be conducted using mixed linear models, including repeated measures models. An appropriate structure for the covariance matrix (e.g., the unstructured covariance matrix) will be selected for these models using the final data. When a model term is statistically significant, the Tukey–Kramer multiple comparisons test will be used to determine which specific pair of means are significantly different. This method will allow us to compare changes over time (within-group changes) and differences between groups simultaneously. Covariates (e.g., length of enteral and solid feeding and length of stay in acute and rehabilitation care) will be accounted for in these analyses. Overall cross-sectional between-group comparisons, such as baseline comparisons used to examine pretest parameters, may be performed using the two-group *t* test, and overall within-group comparisons may be performed using the paired *t* test. If assumptions of normality of distribution for the above tests are not tenable, variables may be transformed prior to analysis, or appropriate nonparametric tests, such as the Wilcoxon rank-sum and signed-rank tests, may be used. Statistical tests will be two-sided and will be performed using a 5% significance level. Stratified analyses accounting for different injury levels will also be performed. These analyses will be specific to the distribution of data in each individual subgroup, which will minimize heterogeneity and allow us to analyze subgroups that may be more statistically homogenous and clinically relevant. SAS software, version 9.4 or later, will be used to conduct the statistical analyses. Multiple imputation methods will be used to address missing data for variables with moderate amounts of missing data (≥10%) and after examining whether data are missing completely at random, missing at random, or missing not at random. Missing data analysis will not be performed on variables with <10% missing data to avoid statistical bias larger than that obtained through the use of complete case analysis.

For microbiome analysis, alpha diversity indices will be analyzed using linear mixed-model with time (pre- and post-) and group (KD and SD) as fixed factors and participant as a random effect. Permutational multivariate analysis of variance testing will be used to assess the effects of time, treatment, and time × treatment on beta diversity indices. The principal coordinate analysis will be performed to visualize the beta diversity distance matrices [18].

For targeted proteomics, three specific analytic approaches will be utilized. First, two-sample *t* tests, or Wilcoxon rank-sum tests if the normality assumption is questionable, will be used to examine differences in the number of selected peptides and proteins between diet groups at discharge (approximately 5 weeks). Because of the number of proteins to be examined, a false discovery rate of 0.2 will be used to account for multiple hypothesis tests. The second approach will examine the manner in which levels of selected peptides vary across the longitudinal time points of baseline, rehabilitation and discharge. To examine these patterns, mixed linear models such as repeated measures models using time as a within-person factor and diet as a between-person factor will be constructed. Finally, Pearson’s correlation will be used to measure the strength and direction of linear associations between selected peptide levels and 5-week outcomes of safety, neurological, functional and glycemic status. 

### Trial monitoring

No regular external trial auditing is scheduled. However, the Data and Safety Monitoring Committee (DSMC) includes two external safety monitors and three study personnel including the PI, study physician and a postdoctoral fellow. Key investigators and study staff provide the DSMC with information regarding safety end points, data quality and validity every 3 months over the course of the study. All human participant data, ranging from recruitment/screening to diet intervention and testing to laboratory tests, are reviewed every 3 months by key study staff, the PI and the DSMC. This includes updates on any new hazards, risks or adverse events, and plans of action. Additional investigators and staff will be asked to participate as the need for their input or expertise arises. If during the course of these meetings particular unforeseen hazards or risks are identified that may predispose to an unusually high number of serious adverse events, the PI consults the appropriate members of the investigative team, as well as the UAB IRB and National Institutes of Health (NIH)/National Institute of Nursing Research (NINR) to determine if the study should be terminated or altered in some way. Any procedure that is deemed hazardous is eliminated from the study and replaced with an alternative if one with reasonable risk can be identified.

### Adverse event monitoring and reporting

#### Reporting

The PI will be responsible for the accurate documentation, investigation and follow-up of all possible study-related adverse events. All adverse events are reported by the PI to the UAB IRB with a description of the event, when and how it was reported, and appropriate documentation to corroborate the event. The description of the adverse event includes all information listed on the case report forms. The IRB then determines whether additional reporting to the NIH/NINR is required.

#### Serious adverse events

All serious adverse events are reported immediately to both the study physician and PI, and then, in turn, reported to the IRB within 48 h. In addition, all serious adverse events that are possibly related to the study intervention are reported to the NIH/NINR within 2 weeks.

#### Nonserious adverse events

Nonserious events are reported to the IRB within 5 business days of the event.

#### Annual reports

An annual statement summarizing the results of safety monitoring is sent to the UAB IRB and to the NIH/NINR. The annual report includes written summaries from quarterly meetings between the DSMC, key personnel and staff, descriptions of all adverse events, and a summary discussing: 1) whether all participants met entry criteria; 2) whether adverse event rates are consistent with expected rates and seriousness; 3) reasons for dropouts from the study; 4) whether continuation of the study is justified; and 5) whether conditions have been met for terminating the study prematurely.

#### Post-trial care

Only minimal risks are anticipated from study participation. Treatment will be provided should adverse events occur but compensation will not be provided for such treatments.

### Protocol amendments

Any change to the protocol requires a written protocol amendment that must be approved by the NIH/NINR and IRB before implementation. Upon acceptance from the sponsor and IRB, the PI makes updates and edits to the study record published on ClinicalTrials.gov. If the PI determines that an immediate change or deviation from the protocol is necessary for safety reasons to eliminate an immediate hazard to the participants, the IRB will be notified immediately.

### Confidentiality

To protect privacy and confidentiality, any original paperwork documenting a participant’s name and PHI is stored in a locked cabinet in the research coordinator’s office, while any digital study records involving PHI are stored in REDCap and/or on computers requiring password authentication that are stored in locked offices and are behind secure firewalls. Records that identify study participants are kept confidential as required by law, and every effort is made to maintain the confidentiality of participants’ study records. Except when required by law or if necessary to protect their rights or welfare, study participants are not identified by name or any other identifying characteristic in records disclosed to those outside of the study staff.

### Access to data

Only study staff, the UAB IRB, official overseers of clinical research at UAB, and representatives of the NIH have access to study records, data and specimens; all access is on a need-to-know basis. All study staff are trained in HIPAA standards for protecting PHI and do not refer to PHI or confidential information in the presence of individuals outside of the study team. Moreover, each study staff member’s access to participants’ data is limited to only the functions for which they are responsible.

### Dissemination policy

The results of our research will be disseminated to three major stakeholders: a) the scientific community; b) individuals with SCI; and c) the general public.

#### a) Scientific Community

The results of this project will be presented at scientific conferences, including at the American Congress of Rehabilitation Medicine, the American Spinal Injury Association and the Society for Neuroscience annual meetings. In addition, results will be published in peer-reviewed journals with open access policies and/or in top-tier journals. The timeline for this is up to 18 months after the last participant completes the study intervention.

#### b) Individuals with SCI

With a timeline of up to 12 months after the last participant completes the study intervention, information will be disseminated to individuals with SCI using the following means.

First, via the UAB Spinal Cord Injury Model System (SCIMS) Information Network website. This website, which averages more than 43,000 visits per year, is newly designed and features a comprehensive collection of links (over 360 to date) to SCI-related information provided by reputable organizations, associations and educational institutions, including from the Model Systems Knowledge Translation Center and other SCIMS centers. The site will be regularly updated with nutrition information and links to other reliable sites of interest and value to SCI consumers. All educational materials written and produced from this project will be made available free on this website.

Second, the UAB-SCIMS newsletter (*Pushin’ On*; now a fully digital newsletter) has been published for 33 years. It provides persons with SCI and their families with information of interest. Over the years, the newsletter has featured original articles along with news, information and synopses of research of importance to individuals with SCI. The study team will work with the newsletter editor to write a synopsis of the proposed research trial, including a description of the diet intervention and a lay summary of major findings and how to implement them.

Finally, for more than 60 years, *Paraplegia News* has been a leader in the wheelchair community for pertinent practical news and information. Paraplegia News Online is an extension of *Paraplegia News* that is easily accessible on the worldwide web and that is dedicated to bringing the very best of real-time, up-to-the-moment news and information for wheelchair users, family members and medical professionals on the go. The study team will work with the newsletter editor to write a synopsis of the proposed research trial including the description of diet intervention and a lay summary of major findings and how to implement them.

#### c) General public

Study findings will be communicated to the general public by collaborating with journalists. The PI will reach out to several of her contacts in radio, television and the print media with a plan to write a feature article on the proposed project. This has a timeline of up to 18 months after the last participant completes the study intervention.

## Discussion

Despite extensive research, no pharmacologic therapy has demonstrated significant improvement on the neurological or functional recovery in people with SCI, and the need for innovative therapies remains urgent. The scientific premise of this project rests on accumulating evidence that diet-based therapies, such as the KD, offer effective neuroprotection against secondary injury cascades and improve forelimb motor function in a rat model of SCI and improve upper extremity motor function in patients with acute SCI. The KD is a high-fat, low-carbohydrate diet designed to mimic the metabolic and biochemical changes that occur during calorie restriction, specifically ketosis. Ketone bodies have been shown to exert neuroprotective effects by preventing oxidative damage, attenuating neuroinflammation and glutamate excitotoxicity and inhibiting apoptosis in the brain and spinal cord. Because glutamate excitotoxicity, inflammation and induction of apoptotic pathways lead to progressive degeneration in the spinal cord shortly after the injury, inhibition of these processes by ketone bodies may enhance neurological recovery after an SCI. In support of these hypotheses, we recently showed for the first time that, compared with an SD, 5 weeks of a KD improved upper extremity motor function in patients with acute SCI (Fig. 1) [20]. In addition, a neuroinflammatory blood protein, fibrinogen, was present at lower levels in the KD serum samples than in the SD serum samples. Taken together, our preliminary results suggest that a KD may induce anti-inflammatory effects in part by reducing fibrinogen, which promotes neuroprotection and improved recovery.

On the basis of these preliminary data in patients, we have designed a randomized controlled trial to examine the effects of a novel nutritional intervention, the KD, on improving sensory and motor recovery, functional independence and glycemic control in patients with acute SCI and on the molecular mechanisms thereof. Our findings may lead to a safe, effective, simple and economical intervention that improves health outcomes for these patients. Changing patients to a more neuroprotective diet during acute care of SCI can be implemented anywhere in the world at low cost and without major regulatory hurdles. Better functional recovery will lead to a better quality of life and long-term health outcomes in individuals with SCI. Therefore, the risks incurred by participants in the study are minor relative to the benefit of improving the quality of life of individuals with SCI.

### Trial status

The study has been active and open for enrollment since September 2019. Enrollment is expected to be completed in December 2023. The clinical trial number is NCT03509571.

## Supplementary information

**Additional file 1.** Standard Protocol Items: Recommendations for Interventional Trials (SPIRIT) checklist.

## Data Availability

The datasets used and/or analyzed during the current study will be available from the corresponding author on reasonable request.

## Abbreviations

AIS: American Spinal Injury Association Impairment Scale
ANCOVA: Analysis of covariance
DSMC: Data and Safety Monitoring Committee
ddH_2_O: Double-distilled water
EPT: Electrical perception threshold
ESI: Electrospray ionization
IRB: Institutional Review Board
ISNCSCI: International Standards for Neurological Classification of SCI
KD: Ketogenic diet
LC: Liquid chromatography
MS: Mass spectrometry
NIH: National Institutes of Health
NINR: National Institute of Nursing Research
PHI: Protected health information
PI: Principal investigator
REE: Resting energy expenditure
SCI: Spinal cord injury
SCIM: Spinal Cord Independence Measure
SD: Standard hospital diet
SPIRIT: Standard Protocol Items: Recommendations for Interventional Trials
SRC: Spain Rehabilitation Center
UAB: University of Alabama at Birmingham
